# Interleukin‐6 receptor alpha blockade improves skin lesions in a murine model of systemic lupus erythematosus

**DOI:** 10.1111/exd.12934

**Published:** 2016-04-26

**Authors:** Peter Birner, Susanne Heider, Peter Petzelbauer, Peter Wolf, Christoph Kornauth, Madeleine Kuroll, Olaf Merkel, Günter Steiner, Tadamitsu Kishimoto, Stefan Rose‐John, Afschin Soleiman, Richard Moriggl, Lukas Kenner

**Affiliations:** ^1^ Department of PathologyMedical University of ViennaViennaAustria; ^2^ Ludwig Boltzmann Institute for Cancer ResearchViennaAustria; ^3^ Department of DermatologyMedical University of ViennaViennaAustria; ^4^ Department of Dermatology and VenereologyMedical University of GrazGrazAustria; ^5^ Division of RheumatologyDepartment of Internal Medicine IIIOsaka UniversitySuita CityOsakaJapan; ^6^ Laboratory of Immune RegulationGraduate School of Frontier BiosciencesOsaka UniversitySuita CityOsakaJapan; ^7^ Institute of BiochemistryUniversity of KielKielGermany; ^8^ Labor Dr. SoleimanHallAustria; ^9^ Institute of Animal Breeding and GeneticsUniversity of Veterinary Medicine ViennaViennaAustria; ^10^ Medical University of ViennaViennaAustria; ^11^ Institute of Laboratory Animal PathologyUniversity of Veterinary Medicine ViennaViennaAustria

**Keywords:** interleukin 6, jun B, SLE

## Abstract

Systemic lupus erythematosus (SLE) is an autoimmune disease, characterized by antinuclear autoantibodies (ANA) and immunocomplexes, commonly affecting kidneys, skin, heart, lung or even the brain. We have shown that JunB^Δep^ mice develop a SLE phenotype linked to increased epidermal Interleukin (IL)‐6 secretion. Blocking of IL‐6 receptor alpha (IL‐6R*α*) is considered as therapeutic strategy for the treatment of SLE. JunB^Δep^ and wild‐type mice were treated for short (5 weeks) or long term (21 weeks) with the IL‐6R*α*‐blocking antibody MR16‐1. Skin and kidney of mice were investigated by histology and immunofluorescence, and in addition, kidneys were analysed by electron microscopy. Furthermore, soluble IL‐6R (sIL‐6R), antihistone and antinucleosome antibodies levels were measured and associated with disease parameters. Treatment with MR16‐1 resulted in significant improvement of SLE‐like skin lesions in JunB^Δep^ mice, compared to untreated mice. The sIL‐6R amount upon long‐term treatment with MR16‐1 was significantly higher in JunB^Δep^ versus untreated JunB^Δep^ (*P* = 0.034) or wild‐type mice (*P* = 0.034). MR16‐1 treatment over these time spans did not significantly improve kidney pathology of immunoglobulin deposits causing impaired function. Significantly higher antihistone (*P* = 0.028) and antinucleosome antibody levels (*P* = 0.028) were measured in MR16‐1‐treated JunB^Δep^ mice after treatment compared to levels before therapy. In conclusion, blockade of IL‐6R*α* improves skin lesions in a murine SLE model, but does not have a beneficial effect on autoimmune‐mediated kidney pathology. Inhibition of IL‐6R signalling might be helpful in lupus cases with predominant skin involvement, but combinatorial treatment might be required to restrain autoantibodies.

## Introduction

Systemic lupus erythematosus (SLE) is a generalized autoimmune disease with an annual incidence of up to 4.5 per 100 000 people at risk and a prevalence of about 50 per 100.000 population 1, 2. The disease is characterized by antinuclear autoantibodies (ANA) and formation of immunocomplexes, affecting a variety of tissues 3, 4. Untreated or insufficiently treated SLE might result in severe complications or death of patients. Today's state‐of‐the art therapy involves livelong immunosuppression, but it might not prevent organ damage, despite problematic side effects 5, 6.

Although many organs can be affected by SLE, the skin and kidneys are most frequently affected by the disease. Untreated or insufficiently treated SLE might result in severe complications or death of patients. Today's state‐of‐the art therapy such as livelong immunosuppression, antimalarials or belimumab might not prevent organ damage, and it is associated with problematic side effects 5, 6. Therefore, a strong need for novel disease‐specific therapies of SLE exists. Increased levels of interleukin‐6 (IL‐6) have been observed in SLE, and IL‐6 signalling is thought to play an important pathogenetic role 7, 8, 9, 10. Recently, anti‐IL‐6R antibodies have turned into the centre of interest for the treatment of autoimmune diseases such as rheumatoid arthritis (RA), systemic onset juvenile idiopathic arthritis (JIA), adult Still's disease and Castleman's disease 11. Furthermore, IL‐6R antibodies have also been suggested for the treatment of human SLE 12. Therefore, the aim of our study was to evaluate the effects of an IL‐6R*α* blocking antibody (MR16‐1) in a murine JunB^Δep^ transgenic model of SLE.

## Material and methods

### Animals

JunB^Δep^ mice were generated as described previously 10. In brief, mice harbouring a floxed JunB allele were generated by targeted homologous recombination. For conditional deletion of JunB in the skin, JunB^f/f^ mice were crossed to K5‐Cre2 transgenic mice. The genetic background of JunB^f/f^ and K5Cre2 mice was C57BL/6/129SV. Mice were genotyped by PCR. All of the procedures were approved by the local Animal Care and User Committees of the Austrian government and the Medical University Vienna (MWF‐66.009/0282‐II/3b/2012). Mice were monitored daily and housed with alternating 12‐albumin light and dark cycles under specific pathogen‐free conditions according to the guidelines of the Medical University of Vienna. All efforts were made to minimize potential animal suffering.

### Blocking of IL‐6R*α*

IL‐6R blocking was performed using the antibody MR16‐1 (provided by Dr. Masahiko Mihara, Chugai Pharmaceutical Co. Ltd., Tokyo, Japan) 13. MR16‐1 binds to mouse‐soluble IL‐6R with a K_D_ value of approximately 10 nmol/l. The antibody was purified using protein G columns. MR16‐1 was administered at a dosage of 8 mg/kg, in 200 *μ*l in PBS i.p. once a week for short (5 weeks) or long time (21 weeks). Treatment started when first SLE‐like skin symptoms became evident at the age of 3 months. Mice were sacrificed at the end of the treatment period, and tissue samples were stored for further analysis. In particular, ten JunB^Δep^ mice were treated with MR16‐1 (6 for 5 weeks, 4 for 21 weeks) and seven JunB^Δep^ mice served as control that received only PBS (4 for 5 weeks, 3 for 21 weeks). As a comparison, ten wild‐type mice were treated with MR16‐1 for 5 weeks, and three wild‐type mice served as control without therapy.

### Microscopic evaluation of treatment response

For the detection of IgG deposits in the epidermal–dermal junction, direct immunofluorescence with goat anti‐mouse IgG (ALBUMIN+L) antibody (Alexa Fluor 488, 1:1000; Invitrogen, Carlsbad, CA, USA) was performed using standard protocols on frozen material.

For histological evaluation of skin lesions, a new semi‐quantitative scoring system (skin score) was developed: thickness of the epidermis, epidermal hyperkeratosis, dermal mucinosis (assessed by Alcian blue staining) and inflammatory infiltrates were each scored from 0 (absent), 1 (mild), 2 (medium) to 3 (severe). Subsequently, the scores were added, resulting in a total score, ranging from 0 to a maximum of 12. For electron microscopy, small pieces of kidney tissue (2 mm diameter) were fixed in 4% paraformaldehyde, 0.1% glutaraldehyde in cacodylate buffer (pH 7.3), and embedded in Epon. Ultrathin sections were stained with uranyl acetate and lead citrate and examined in a Jeol 105 electron microscope.

### Detection of autoantibodies and ELISA

Antihistone and antinucleosome IgG antibodies were measured at week 5 or 21 by ELISA (Inova Diagnostics, San Diego, CA, USA), using horseradish peroxidase‐conjugated anti‐mouse antibodies (Southern Biotech, Birmingham, AL, USA 1:2000) as secondary antibodies 14. Additionally, sera were analysed by line immunoassay (Fujirebio Diagnostics, Göteborg, Sweden) for the presence of antibodies against the antigens SmB, SmD, U1‐70K, U1‐A, U1‐C, Ro60. Ro52 (TRIM21), La, topoisomerase I, Jo1, Centromere Protein B, and Ribosomal P protein as previously described 10. Serum levels of sIL‐6R were determined by ELISA (DY1830, R&D Systems, Minneapolis, MN, USA). Urine albumin levels were investigated using a murine *α*‐Albumin ELISA quantitation Kit (Cat No.: E90‐134, Bethyl Lab.Inc., Montgomery, TX, USA) at beginning and end of treatment.

### Statistics

Wilcoxon tests, Mann–Whitney *U*‐test, Kruskal–Wallis and chi‐square tests and logistic regression were used as appropriate. SPSS 20.0 was used for all calculations. A two‐tailed *P*‐value of equal or <0.05 was considered as significant. Numbers given are mean values ± standard deviation if not stated otherwise.

## Results

### Phenotypic organ alterations

**Figure 1:**
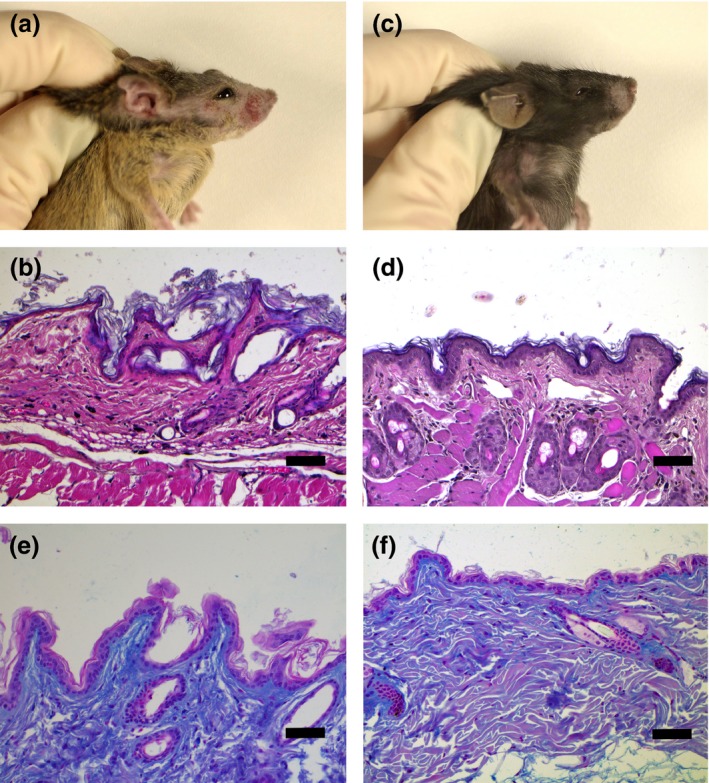
(a) JunB^Δep^ mouse without therapy after 21 weeks. Note the dermatitis on the head of a representative mouse. Sharply demarcated, erythematous, confluent patches and scattered flat papules were present on the lateral parts of snout and in the periocular and auricular region. (b) Histological specimen of snout skin of a JunB^Δep^ mouse without therapy: Note the thin, atrophic, epidermis with distinct hyperkeratosis and slight thickening of the basement membrane and vacuolation at the dermo‐epidermal junction. Hair follicles were lacking. Inflammatory infiltrates are absent (H&E, original magnification x200, bar represents 100 *μ*m). (c) Picture of a JunB^Δep^ mouse which received MR16‐1 therapy after 21 weeks. Note the significantly less pronounced lesions of the snout compared to the untreated JunB^Δep^ animal shown in Fig. 1a. The different fur colour compared to the mouse shown in Fig. 1a is a consequence of mixed background C57bL/6/129Sv. Thus, the outbred mouse colony contained black, agouti and white animals. (d) Histological specimen of snout skin of a JunB^Δep^ mouse with MR16‐1 therapy after 21 weeks: In contrast to Fig. 1b, normal appearance of the skin at histological investigation is clearly visible (H&E, original magnification x200, bar represents 100 *μ*m). (e) Alcian blue‐stained skin section of a JunB^Δep^ mouse without therapy after 21 weeks: Note the discrete hyperkeratosis and the dermal mucin staining (blue colour; Original magnification x200, bar represents 100 *μ*m). (f) Alcian blue‐stained skin section of a JunB^Δep^ mouse which received MR16‐1 therapy after 21 weeks: Note that dermal mucinosis is absent (Original magnification x200, bar represents 100 *μ*m).

All JunB^Δep^ mice examined developed a characteristic dermatitis of ears, snouts, upper thorax region and paws starting at the age of 3 months postpartum without exposure to UV light (Fig. 1). Histology revealed an atrophic, thin epidermis with hyperkeratosis and slight thickening of the basement membrane and vacuolation at the dermo‐epidermal junction. Mucin deposits were present in the dermis, together with pilosebaceous atrophy.

Kidney lesions were characterized by mesangial hypercellularity and basement membrane thickening with lobulation of the glomerular tuft resembling an immunocomplex glomerulonephritis (IC‐GN). Most of the lesions also showed massive features of endocapillary hypercellularity, and luminal obstruction by immunocomplex deposits. These lesions were also described in further detail in our previous paper where we described the SLE animal model of JunB^Δep^ mice 10. In line with earlier findings, systemic SLE pathology (including kidney affection) is not evident in the first weeks of life, but develops upon ageing in JunB^Δep^ mice 10.

### Effects of MR16‐1 treatment on skin and kidney lesions

After treatment with MR16‐1, a significant improvement of skin lesion was observed in JunB^Δep^ mice. During the 5‐week treatment period of the first group of treated mice, the extension of lesions decreased and the thickness of the epidermis normalized. Skin score was significantly higher in untreated mice compared to treated ones, irrespectively of duration of therapy (median 5 vs 1; *P* = 0.002, Mann–Whitney *U*‐test). In contrast, all wild‐type mice showed no SLE‐like symptoms and administration of MR16‐1 was well tolerated. In light microscopy, no changes in the skin or the kidneys were evident, and no difference in weight after 5 weeks of treatment between treated wild‐type mice and all other groups was observed (*P* > 0.05, Kruskal–Wallis test). No mesangial deposits were observed in the kidneys of a treated JunB^Δep^ mouse, while discrete deposits were seen in 5 and moderate deposits in 4 treated JunB^Δep^ mice.

Immunofluorescence (IF) for IgG deposits in the epidermal–dermal junction was performed in 14 JunB^Δep^ mice, comprising 8 mice of the 5‐week group (4 treated, 4 untreated) and 6 animals of the 21‐week group (3 untreated, 3 treated). Interestingly, in the 5‐week group, in 3 of 4 treated mice faint epidermal/dermal IgG deposits were seen, but in none of the 4 untreated animals. In the 21‐week group, no treated mouse showed IgG deposits, but 2 of 3 of untreated mice had prominent IgG deposit features (Table 1). Despite this clear trend of disease change with improved skin pathology, no significance was found (*P* > 0.05, chi‐square test), most probably due to the low number of animals in each subgroup. In the 10 treated wild‐type mice, no immunodeposits were found irrespective of treatment.

**Table 1:** Parameters investigated in mouse groups (median values and range, if not otherwise indicated)

	Group of JunB^Δep^ mice			
	Untreated 5 weeks	Untreated 21 weeks	MR16‐1 5 weeks	MR16‐1 21 weeks
Urine albumin (mg/l) start	3.1 (2.7–4.9)	8.8 (5.9–38)	4.2 (1.4–7.1)	6 (5.1–40)
Urine albumin (mg/l) end	1.9 (0.6–32)	8.7 (7.7–39)	3.5 (1.5–4.9)	14.6 (5.6–17.1)
Antihistone antibodies start^a^	19 (19–32)	35 (29–45)	19 (19–63)	29 (9–40)
Antihistone antibodies end^a^	30 (19–64)	39 (34–100)	34 (19–100)	100 (100–100)
Antinucleosome antibodies start^a^	19 (19–19)	24 (16–100)	19 (19–24)	30 (7–34)
Antinucleosome antibodies end^a^	19 (19–27)	26 (24–62)	19 (19–76)	100 (100–100)
Soluble IL‐6R (pg/ml) end	n.d.	2233 (2132–2432)	n.d.	4364 (3973–5329)
Skin score	5.5 (2–7)	5 (2–6)	1 (0–2)	1.5 (0–3)
Number (percentage) of mice with epidermal–dermal IgG deposits	0/4	2/3 (66.7%)	3/4 (75%)	0/3
Body weight (g) start	23 (17–27)	24 (21–25)	23 (19–28)	20 (16–22)
Body weight (g) end	21 (17–24)	21 (16–25)	24 (20–32)	20 (16–24)

a ‘19’ indicates no detectable levels, ‘100’ is the possible relative maximum at the used ELISA.

### Electron microscopy of kidneys

Electron microscopic analysis of kidneys of 10 treated mice in total was performed; six mice for a 5‐week period, four mice for a 21‐week period and 6 untreated control mice. No subendothelial immune complex deposits were found in any of the treated or untreated mice. Subepithelial immune complex deposits were found in only one treated mouse (21 weeks), mesenchymal deposits in all but one (5 weeks) and all but one (21 weeks) untreated mice.

### Effect of MR16‐1 treatment on body weight

The body weight of the mice started to increase in the treated JunB^Δep^ mice after 5 weeks compared to untreated ones (plus 2 ± 1.4 g in treated vs minus 1.5 ± 1.7 g in untreated mice; *P* > 0.05, Wilcoxon test). To investigate whether a longer treatment with MR16‐1 would further improve the disease in JunB^Δep^ mice, a second cohort of mice was treated for 21 weeks. During the longer period of MR16‐1 treatment, JunB^Δep^ mice improved significantly regarding the skin phenotype (Fig. 1); however, no further increase of body weight could be measured compared to JunB^Δep^ mice of the treated 5‐week cohort (*P* > 0.05, Wilcoxon test). No significant difference in weight at the start of the observation period and at the end of week 5 or week 21, respectively, was seen (*P* > 0.05, Wilcoxon test).

### The impact of MR16‐1 treatment on urine Albumin levels was negligible

**Figure 2:**
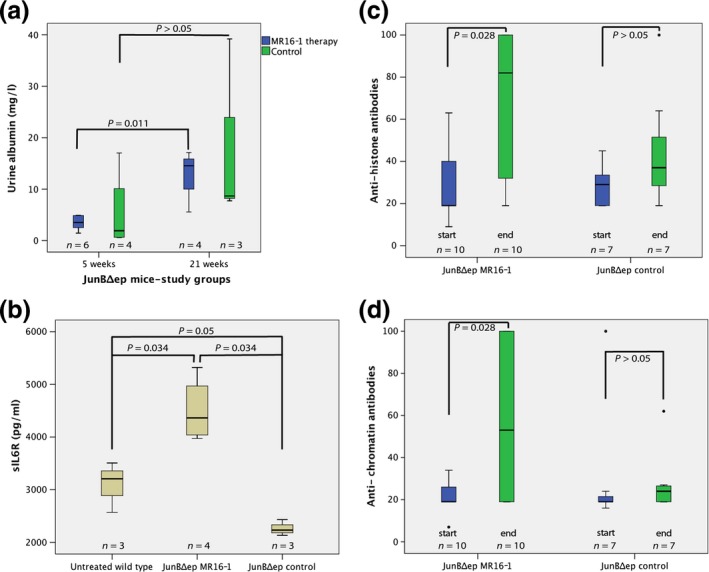
(a) Urine albumin levels (mg/l) in subgroups at 5 and 21 weeks of MR16‐1 treatment at the end of the study period. (b) sIL‐6R levels (pg/ml) at the end of observation period/treatment in subgroups. Note the significant increase in sIL‐6R levels in the MR16‐1‐treated animals (*P* = 0.034). (c) Antihistone antibody levels (arbitrary units) at the start and at the end of the observation period (5 or 21 weeks, respectively). Note the significantly increased antibody levels in the therapy group (*P* = 0.028). (d) Antinucleosome antibody levels (arbitrary units) at the start and at the end of the observation period (5 or 21 weeks, respectively). Note the significantly increased antibody levels in the therapy group (*P* = 0.028).

Generally, urine Albumin levels were higher in the 21‐week group compared to the 5‐week group in MR16‐1‐treated JunB^Δep^ mice (*P* = 0.011, exact Mann–Whitney *U*‐test, Table 1). Similar trends towards higher urine Albumin levels in the 21‐week vs the 5‐week groups were seen in untreated JunB^Δep^ mice, but this did not reach significance (*P* > 0.05, Mann–Whitney *U*‐tests, respectively). When comparing urine Albumin levels before and after MR16‐1 treatment, no significant difference between all animals or the 5‐ and 21‐week treatment groups was found, irrespectively of observation period (*P* > 0.05, Wilcoxon tests). In addition, no difference in urine Albumin levels at the end of the study period was found between groups, irrespectively of therapy length (*P* > 0.05, Kruskal–Wallis tests) (Fig. 2a).

**Figure 3:**
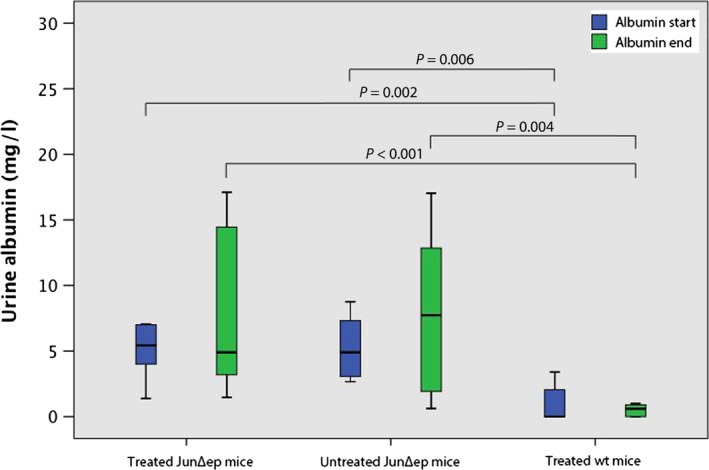
Urine albumin levels (mg/l) in treated and untreated mice at the beginning (‘Albumin start’) and at the end (‘Albumin end’) of the study.

We found also no difference between albumin levels before and after MR16‐1 therapy in wild‐type mice (*P* > 0.05, Wilcoxon test). Significant lower albumin levels in wild‐type mice that received MR16‐1 compared to treated JunB^Δep^ mice were seen before (median 0, range 0–10.3 mg/l vs 5.43, range 1.39–40.3 mg/dl; *P* = 0.002, Mann–Whitney *U*‐test) and after treatment (median 0.6, range 0–3.1 mg/dl vs 4.9, range 1.46–17.11; *P* < 0.001, chi‐square test) (Fig. 3). Albumin levels were also significantly lower in treated wild type compared to untreated JunB^Δep^ mice before (median 0, range 0–10.3 mg/dl vs 4.9, range 2.66–38 mg/dl, *P* = 0.006. Mann–Whitney *U*‐test) and after therapy (median 0.6, range 0–3.1 mg/dl vs 7.73, range 0.62–39.2, *P* = 0.004, Mann–Whitney *U*‐test) (Fig. 3).

### MR16‐1 treatment increases sIL‐6R and autoantibody levels

Data on sIL‐6R levels were only available from the 21‐week group. A significant difference in sIL‐6R levels was seen between groups (*P* = 0.024, Kruskal–Wallis test). Subsequent pairwise comparisons revealed that sIL6R levels were significantly higher in JunB^Δep^ mice treated with MR16‐1 compared to untreated ones (*P* = 0.034, Mann–Whitney *U*‐test, Table 1) or untreated wild‐type mice (*P* = 0.034, Mann–Whitney *U*‐test). The sIL‐6R levels were also lower in JunB^Δep^ mice without treatment compared to untreated wild‐type mice (*P* = 0.05, respectively, chi‐square test) (Fig. 2b).

When comparing subgroups according to treatment irrespective of treatment duration, significant higher antihistone (*P* = 0.028, Wilcoxon test, Fig. 2c, Table 1) and antinucleosome antibody levels (*P* = 0.028, Wilcoxon test, Fig. 2d, Table 1) were observed at the end compared to the beginning of the observation period, but only in MR16‐1‐treated JunB^Δep^ mice. When autoantibody levels between treated and untreated mice in the 5‐ and 21‐week groups were analysed separately, no significance was reached in the 5 weeks group. In the 21‐week group significance was missed at investigation of antihistone antibodies (*P* = 0.078, Mann–Whitney *U*‐test), and antinucleosome autoantibodies were significantly higher in treated mice (*P* = 0.019, Mann–Whitney *U*‐test) (Table 1). Antihistone antibodies and antinucleosome antibody levels before and after end of the observation period were not different between subgroups (*P* > 0.05, Kruskal–Wallis test). Interestingly, levels of antinucleosome antibodies but not antihistone antibodies were associated with duration of therapy (*P* = 0.021, Mann–Whitney *U*‐test). In a regression model, subgroups (coefficient of regression: ‐22.8, *P* = 0.003) and duration of observation period (coefficient of regression: 27.1, *P* = 0.024) but not levels of antihistone antibodies at the beginning of the observation period influenced antihistone antibody levels at the end of the observation period.

With regard to antinucleosome antibodies, subgroups (coefficient of regression: −20.4, *P* = 0.003) and duration of observation period (coefficient of regression: 36, *P* = 0.003) but not levels of antinucleosome antibodies at the beginning of the observation period influenced antibody levels at the end of the observation period.

Of note, other autoantibodies associated with SLE or other connective tissue diseases, such as anti‐Sm or anti‐Ro antibodies, were not significantly detected in the sera of these mice. No significant correlation could be drawn between sIL‐6R and albumin levels, regardless of the genetic or treatment subgroup.

In conclusion, IL‐6R inhibition improved the SLE skin pathology, but autoimmune‐mediated disease processes in JunB^Δep^ mice cannot sufficiently be cured by this.

## Discussion

IL‐6 has been proposed to play a major a role in the development of SLE and especially in patients with lupus nephritis, IL‐6 levels were found to be increased 1, 2, 5, 6, 7, 8. We have shown in a previous study that mice with loss of epidermal JunB mice develop a SLE phenotype linked to increased epidermal IL‐6 secretion 10. These mice develop ANA, an immunocomplex glomerulonephritis (IC‐GN), albuminuria and lupuslike skin lesions, which could be enhanced by exposure to UV light. The conditional, keratinocyte‐specific (K5Cre) deletion of JunB in our mouse model resulted also in a selective and reproducible secretion of IL‐6 by keratinocytes.

The pathologic effect of epidermal loss of JunB was almost completely rescued in mice after loss of IL‐6, emphasizing the importance of the JunB/IL‐6 axis for the development of SLE. We were also able to show that human SLE patients show reduced epidermal JunB levels associated with high IL‐6 receptor (IL‐6R)‐*α* expression levels 10. These data suggested that our transgenic mouse model might serve as model for human SLE and that blocking of IL‐6R would be a promising therapeutic strategy in SLE 15.

Several IL‐6R blocking antibodies were developed and tested in the clinic, partly due to an important role of inflammatory IL‐6 action in a variety of other human autoimmune diseases 11, 16. IL‐6R inhibition by the monoclonal antibody tocilizumab 17 was recently shown to be beneficial for patients with rheumatoid arthritis 18, 19. Although IL‐6R blockade results in upregulation of IL‐6 levels 20, this regimen is feasible also as a long‐time therapy, as recently shown in human rheumatoid arthritis patients 18. A first clinical phase‐I study on tocilizumab in human SLE showed a decrease in disease activity, warranting further investigations 21. In addition, a case of a SLE patient without response to conventional therapy was reported recently who responded favourably to tocilizumab with remission of fever, arthritis and skin manifestations 22.

MR16‐1 is a well‐established rodent anti‐IL‐6R antibody, binding specifically to mouse IL‐6R. This antibody has been shown to inhibit IL‐6‐induced proliferation of 7TD1 myeloma cells in a dose‐dependent manner. Moreover, this inhibitory effect is reversed by increased IL‐6 concentration 13, demonstrating competition with cytokine‐induced IL‐6 signalling. In addition, MR16‐1 suppressed IL‐6‐induced antibody production 20, and it prevented or improved dermal sclerosis in a murine model of scleroderma 23. Intriguingly, in our study antinucleosome autoantibodies were significantly higher in treated mice compared to controls (Table 1). Said so, antinucleosome antibodies show similar sensitivity and specificity as anti‐dsDNA antibodies, which are considered as a specific serological hallmark of SLE. Indeed, they are viewed as a subset of antinucleosome antibodies. Antinucleosome antibodies react with nucleosomes but not with its constituents DNA and can occur before the development of anti‐dsDNA antibodies 24.

Using the MR16‐1 antibody, we were able to show that in a murine model of SLE, blocking of IL‐6R reduced skin manifestations. MR16‐1 treatment therapy reduced the formation of dermal/epidermal IgG deposits after 21 weeks of treatment (although no significance was reached). In contrast, the formation of immune complex deposits in the kidneys and renal function seems not to be influenced by MR16‐1 treatment.

In addition to our previous finding that loss of IL‐6 prevents formation of SLE symptoms in JunB‐deficient mice 10, we here demonstrate that IL‐6 is a main driver for establishment of SLE‐like skin lesions in JunB^Δep^ mice. Although a positive effect of IL‐6R blocking on lupus nephritis in a murine model has been described by another group 15, in our study no significant benefit of IL‐6R blockage on renal function could be demonstrated. Thus, MR16‐1 treatment does not improve kidney function in JunB^Δep^ mice and the increase of urine Albumin after long‐term treatment is simply associated with ageing and an accelerated autoimmune phenotype. In summary, a clear trend towards higher albumin was seen in aged versus young untreated JunB^Δep^ mice, but again it did not reach statistical significance. This finding is also supported by the fact that albumin levels were significantly lower in wild‐type mice compared to transgenic ones, irrespective of therapy.

Nevertheless, the group of Margarete Hibbs reported recently that in another SLE mouse model (Lyn^−/−^ mice), clinical renal function may improve during IL‐6R antibody therapy, despite increased kidney deposits irrespective of treatment time 25.

We anticipated that sIL‐6R levels in JunB^Δep^ mice would be lower than in wild‐type mice, but surprisingly MR16‐1 treatment increased sIL‐6R levels. This finding is explained by the fact that that secreted IL‐6 binds to the sIL‐6R, and the IL‐6/sIL‐6R complex binds to cells, which express gp130 but not IL‐6R. This causes internalization of the IL‐6/sIL‐6R complex. Thus, this is associated with a decrease in sIL‐6R levels in wild‐type mice.

The presence of the MR16‐1 antibody might have two effects: (i) binding of the antibody to the sIL‐6R will increase the half‐life of the sIL‐6R by increasing the molecular weight, as these complexes are also too large for excretion by the kidneys. (ii) The antibody will inhibit binding of IL‐6 to the sIL‐6R. As binding of IL‐6 to the sIL‐6R is a prerequisite of internalization, this event will prevent internalization. The combination of these two effects (increased half‐life and loss of internalization) leads to an increase of steady‐state levels of the sIL‐6R. This is reminiscent of the increase of IL‐6 levels upon treatment of patients with tocilizumab. It was argued that the prevention of binding of IL‐6 to the IL‐6R (membrane bound or soluble) will lead to less internalization causing increased IL‐6 levels 26.

IL‐6R blockade in the JunB^Δep^ SLE model resulted in a significant increase in levels of autoantibodies. This was never reported so far, and no data exist that concern human SLE patients. Interestingly, in a small series of patients with SLE, antihistone and antinucleosome antibody levels increased in the majority of patients after infusion of infliximab, a chimeric antitumor necrosis factor alpha (TNFa) antibody 27. Interestingly, TNFa signalling blockade was effective as short‐term treatment and autoantibody levels returned to baseline levels several months after the end of therapy 27.

In rheumatoid arthritis, IL‐6 blockade using tocilizumab resulted in a decrease in the levels of IgG4 anticyclic citrullinated peptide (CCP) but not of IgG1 anti‐CCP or antinuclear antibodies (ANA) 28. Although our results show that blocking of IL‐6R is a promising therapeutic opportunity in SLE especially with regard to skin symptoms, the association with increased autoantibody levels deserves further studies, as it might hamper the therapeutic usability of IL‐6R blockade in SLE. A small study with 4 ANA‐positive rheumatoid arthritis patients displayed that ANA antibody levels were not affected by IL‐6R blockade 28, but the small patient cohort might not represent a true mirror image of the real picture in SLE, which again demands for further investigation.

Our data indicate that inhibition of IL‐6 represents a promising therapeutic approach in SLE. Improved skin lesions were a result of IL‐6R signalling blockade, but this was negatively associated with an increase of autoantibody levels posing a therapeutic caveat, as kidney pathology was not significantly improved. We suggest that blocking the IL‐6R might be of special benefit for specific patient subsets (i.e. those with predominant skin involvement) or upon combination with other SLE‐targeted drug regimens. Further studies should investigate if topical application of IL‐6R‐blocking agents might be of benefit for SLE skin lesion to avoid systemic side effects such as an increase in autoantibody levels.

## Conflict of interest statement

None declared.

## Author contribution

PB, PP, PW, SR‐J, RM, GS and LK wrote the manuscript; SH, PP, CK, MK, OM, GS, AS, RM and LK performed the research; PB, PP, PW, and LK analysed the data; GS, SR‐J, RM and LK designed the study; and TK contributed essential reagents.
